# Characterization of the Complete Chloroplast Genome and Phylogenetic Implications of *Euonymus microcarpus* (Oliv.) Sprague

**DOI:** 10.3390/genes13122352

**Published:** 2022-12-13

**Authors:** Hongying Li, Mengdi Chen, Zhengbo Wang, Ziyuan Hao, Xiping Zhao, Wenyan Zhu, Longchang Liu, Wei Guo

**Affiliations:** 1 College of Horticulture and Plant Protection, Henan University of Science and Technology, Luoyang 471023, China; 2 Taishan Academy of Forestry Sciences, Taian 271000, China

**Keywords:** *Euonymus microcarpus* (Oliv.) Sprague, chloroplast genome, genetic diversity, phylogenetic analysis

## Abstract

*Euonymus microcarpus* (Oliv.) Sprague, is a species of evergreen shrub of the genus *Euonymus*, family Celastraceae. Here, we extracted the genomic DNA from the leaves of *E. microcarpus* and constructed a paired-end library. The chloroplast genome of *E. microcarpus* was generated with the high-throughput sequencing by the illumina Hiseq X Ten platform and de novo assembly. The chloroplast genome had a quadripartite structure, containing a long single copy region with a size of 85,386 bp and a short single copy region with a size of 18,456 bp, separated by two inverted repeat regions of 26,850 bp. The chloroplast genome contained 133 genes identified in total, including 87 potential protein-coding genes, 38 transfer RNA genes, and eight ribosomal RNA genes. A total of 282 simple sequence repeats and 63 long repeats were found. Furthermore, the phylogenetic relationships inferred that *E. microcarpus* is sister to *E. japonicus* and *E. schensianus*. A comparison of the structure of the chloroplast genomes of eight *Euonymus* species suggests a nucleotide variability of the junction sites and a higher divergence of non-coding regions, compared to the coding regions. The original findings of the study serves as a good reference for chloroplast genome assembly and a valuable foundation for the genetic diversity and evolution of *E. microcarpus*.

## 1. Introduction

There are about 220 species in the genus *Euonymus*, half of which are distributed in China [1]. The species of the genus *Euonymus* have three fundamental characteristics. The first one is that most of the species in genus *Euonymus* have been used in traditional Chinese herbal medicine for treating tumors, thrombosis, diabetes, hyperlipidemia and inflammation because the herbal extracts contain numerous chemical components, including terpenoids, flavonoids, phenolic acids, lignans, cardenolides and alkaloids [2]. The second one is that most of the species in this genus displays a tolerance to the cold, drought, salt and wounding and are ideal ornamental shrubs or trees [3]. The last one is that the seed oil extracted from the plants in the genus *Euonymus* is composed of 3-acetyl-1,2-diacyl-sn-glycerols, which has a reduced viscosity and a lower crystallization temperature than that of the common vegetable oils and is extremely attractive for industrial purposes [4].

*E. microcarpus* (Oliv.) Sprague, an endemic species in China, distributed in Hubei, Shaanxi, Sichuan and Yunnan provinces, is a species of evergreen shrub of the genus *Euonymus*, family Celastraceae [5,6]. It grows in mixed forests along riversides and on hillsides, at an altitude of 350–1000 m [7]. As an extensively used herb in Chinese traditional medicine, the roots and stems of *E. microcarpus* have been used for relieving the rigidity of muscles, activating the collaterals and relieving rheumatism [7]. As an ideal ornamental plant species, *E. microcarpus* exhibits a tolerance to hard pruning [6].

Chloroplasts, the plastids in plants and green algae originated by the cyanobacteria endosymbiosis, are photosynthetic organelles producing energy for plant growth and development by photosynthesis and the oxygen-release processes [8,9]. More recent research has begun to uncover the novel roles of chloroplasts in plant growth and development. Melatonin produced in chloroplasts and mitochondria is important for plants under stress conditions [10]. The COP1LIKE protein acts as a repressor of photomorphogenesis and plays roles in the reproductive development and light-dependent pigment accumulation [11]. The chloroplast genomes (cpDNAs), maternally inherited in most plant species, are highly conserved in genes fundamental to plant life, gene content and gene order. Typically, cpDNAs are circular, containing a pair of inverted repeats (IRs), a large long single copy (LSC) region and a short single copy (SSC) region [12]. Most of the variations of the cpDNAs in size and structure, caused by plant evolution or special environmental adaptation, can be used to study the genetic diversity, population genetics and phylogenetic relationships among angiosperms [13]. 

The cpDNAs of several species of the genus *Euonymus*, such as *E. phellomanus*, *E. fortunei* and *E. maackii*, were sequenced by high-throughput sequencing and were assembled, annotated and published at present [14]. We collected wild *E. microcarpus* samples for the high-throughput cpDNA sequencing. The genomic features of the *E. microcarpus* cpDNA were determined, then the simple sequence repeats (SSRs) and the long repeats were obtained. The comparisons were made of the cpDNA sequences of *E. microcarpus* and 35 other species within the order Sapindales, then revealed their phylogenetic relationships. 

In the present work, we report the first plastome sequence of *E. microcarpus* to guide the exploration of the use of the new genetic resources and the phylogenetic relationships within the order Sapindales. The findings of this paper provide a foundation for future genomic research on phylogenetic relationships and evolution within the genus *Euonymus*.

## 2. Materials and Methods

### 2.1. Plant materials, Library Construction, and cp Genome Sequencing and Assembly

Fresh and healthy leaves of *E. microcarpus* (Oliv.) Sprague were directly collected by Dr. Hongying Li from an individual at Qiuba, Luanchuan, Luoyang, Henan, China (34°03′58″ N, 111°66′72″ E) and were identified by the most outstanding feature of the colored fall berries that begin shrouded in pink to red capsules and open to orange arils. The voucher specimen (LY_2020_HAUST263) was chilled in a dry ice-ethanol bath, then deposited in a freezer in the Biological Breeding Institute (BBI), Taishan Academy of Forestry Sciences (TAFS), Taian, China (contact person: Dr. Wei Guo, wwwguoweinet@163.com). We extracted the genomic DNA with the CTAB method [15]. The sheared genomic DNA (fragment size ≤ 350 bp) was used for the DNA end repair, A-tailing, adapter ligation, and PCR amplification to construct the paired-end library. The Illumina Hiseq X Ten platform was then employed for sequencing. 

Following the removal of the adapters and the low-quality reads with fastp (version 0.20.0), the read quality was improved and high-quality clean reads were obtained [16]. The *E. microcarpus* cpDNA was assembled using the SPAdes pipeline (http://bioinf.spbau.ru/SPAdes/) (accessed on 3 February 2020) with *E. japonicus* cpDNA as the reference (GenBank accession NC_028067).

### 2.2. Gene Annotation

Once the annotation was completed with the CpGAVAS pipeline, the cpDNA of *E. microcarpus* was deposited to GenBank data libraries under the following accession number ON611716 [17]. A map of the *E. microcarpus* cpDNA was created with OGDRAW (version v1.2) [18]. The program CodonW (version 1.4.4) (http://codonw.sourceforge.net) (accessed on 3 February 2020) was applied for the analyses of the relative synonymous codon usage (RSCU).

### 2.3. Repeats Identification

REPuter was applied to quantify and locate the forward and reverse complement repeats [19,20]. The MISA software (version 1.0) was used to identify the potential SSRs [21].

### 2.4. Phylogenetic Analysis

The alignment of the 36 cpDNAs was conducted with MAFFT (version 7.427) [22]. The phylogenetic trees with a maximum likelihood method were inferred by RAxML (version 8.2.10), using the ‘GTRGAMMA’ evolutionary model, using a rapid boot-strap algorithm of 1000 replications [22].

### 2.5. A Comparison of the Genomes of the Related Species

IRscope was used to visually display and compare the borders of the SSC, IRs and LSC among the eight *Euonymus* species [23]. The mVISTA with the Shuffle-LAGAN program was employed for the pairwise alignment with eight complete cp genomes, using the sequence annotation information of *E. japonicus* [24]. Once the sequences were aligned with the MAFFT (version 7.427), the pi values of the genes were computed using the CDS sequence alignments of the different genes with vcftools, and the Ka/Ks statistic, that is, the ratio of nonsynonymous (Ka) to synonymous (Ks) nucleotide substitution were calculated, based on the alignments with the KaKs_Calculator.

## 3. Results

### 3.1. Genome Characteristics of E. microcarpus

A total of 20,881,158 clean paired-end reads were yielded after discarding the adapters and low-quality reads, which comprised 6,264,347,400 bases, of which the percentage of Q30 was higher than 93.37% and the GC content was 37.55%. The cpDNA assembly of *E. microcarpus*, 157,542 bp in length, has a GC content of 37.22% and an average read coverage of 2321.42×. Compared with the cpDNA of *E. szechuanensis*, that of *E. microcarpus* shared conserved sequences and the synteny was identified (Table S1). 

**Figure 1:**
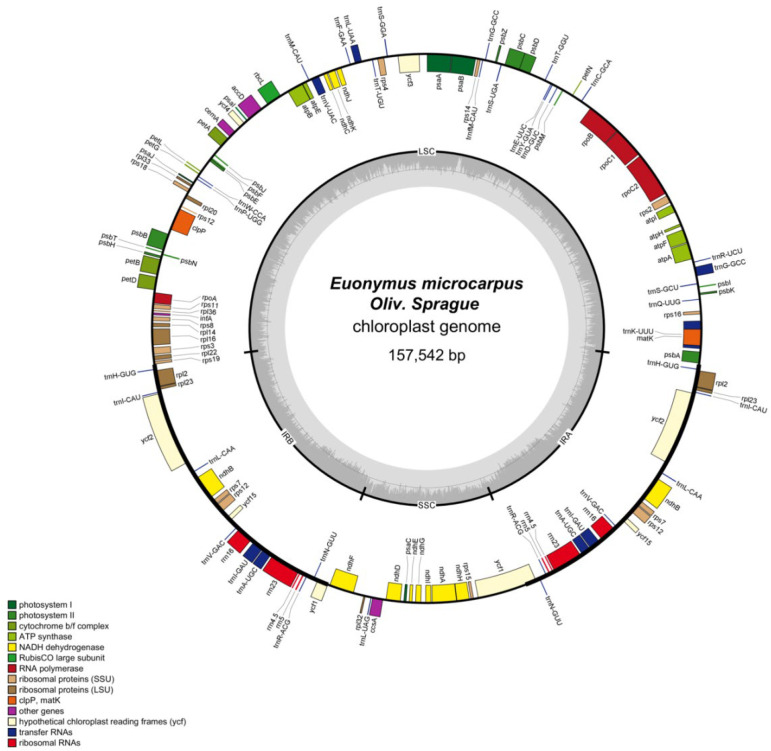
Circular map of the *E. microcarpus* cpDNA. Genes shown outside and inside of the circle map are transcribed clockwise and counterclockwise, respectively. Different colors represent different functional genes. The dashed area in the inner circle corresponds to the GC content.

**Figure 2:**
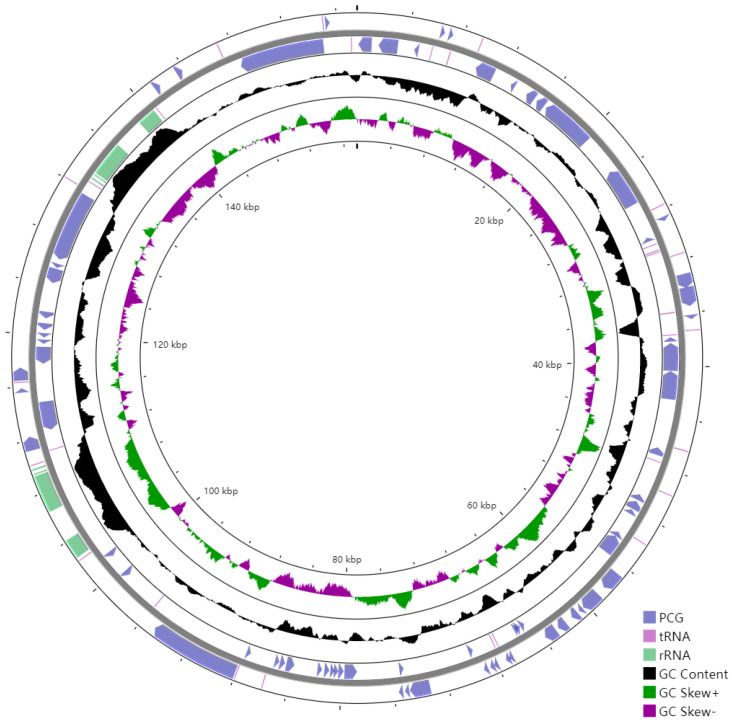
GC content of the *E. microcarpus* cpDNA. From outer into inner, the first and second circles represent the CDSs of the protein coding genes (blue), rRNA operon (light purple), and tRNA information (reddish brown); the third circle represents the GC contents; the fourth circle represents the GC skew [(G − C)/(G + C); green, >0; purple, <0; and the fifth circle represents the coordinates of the cpDNA. The artificial start site is at 0 kbp.

The cpDNA is a circular molecule displaying a characteristic quadripartite structure, containing a LSC region with a size of 85,386 bp and a SSC region with a size of 18,456 bp, separated by two IR regions of 26,850 bp (Figure 1). It was observed that the two IR regions display a considerably higher GC content than that of LSC and SSC regions (Figure 2). 

The cpDNA was predicted to encode 87 potential protein-coding, 38 transfer RNA (tRNA), and eight ribosomal RNA (rRNA) genes (Table 1). In total, the duplication of 17 genes happened in the two IRs, including seven protein-coding genes, *rpl2*, *rpl23*, *ycf2*, *ycf15*, *rps7*, *rps12* and *ndhB* (Table 1). Seventeen intron-containing genes were annotated, 11 of which were protein-coding genes, whereas the remaining six were tRNA genes (Table 2). Among the 11 protein-coding genes identified in this study, *clpP*, *ycf3* and *rps12* bear two introns, while the others bear only one. The *rps12* gene was identified as trans-spliced with a single 5′-end at the LSC region while a repeated 3′-end exons was located in the IRs (Figure 1 and Table 2).

**Figure 3:**
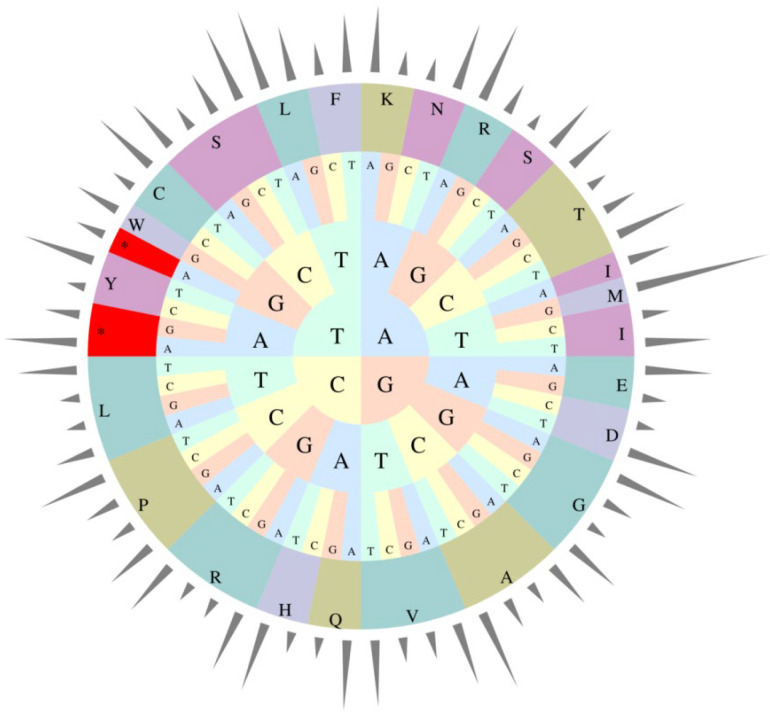
Codon usage frequency of the *E. microcarpus* cpDNA.

The relative occurrence of the synonymous codons in the coding sequences of *E. microcarpus* cpDNA was calculated using 26,883 codons. It suggests that the four most frequently used codons are AAA-K (1114), AUU-I (1093), GAA-E (1058) and UUU-F (982), accounting for 4.14%, 4.07%, 3.94% and 3.65% among all codons, respectively (Table S2 and Figure 3). Similar to the previous results for other angiosperms, most of the codons ending with A or T have RSCU values greater than 1, while, most of those ending with C or G have RSCU values of less than 1 [25,26,27]. 

### 3.2. Analysis of the Repeats

In the cpDNA of *E. microcarpus*, a total of 63 long repeats, ranging from 30 to 77 bp in length, were recognized, including 16 forward, 20 reverse, 6 complement and 21 palindrome repeats (Table S3). Furthermore, a total of 42, 29 and 9 long repeats were detected in the LSC, IRs and SSC regions, respectively.

In addition, 282 simple sequence repeats (SSRs) were detected in the cpDNA, among which 199, 10, 82, 8 and 4 were mono-, di-, tri-, tetra- and pentanucleotide repeats, respectively (Table S4). In all of the SSRs identified in this study, the mononucleotide SSRs were the most abundant, with an abundance of 70.57%.

### 3.3. Phylogenetic Analysis

**Figure 4:**
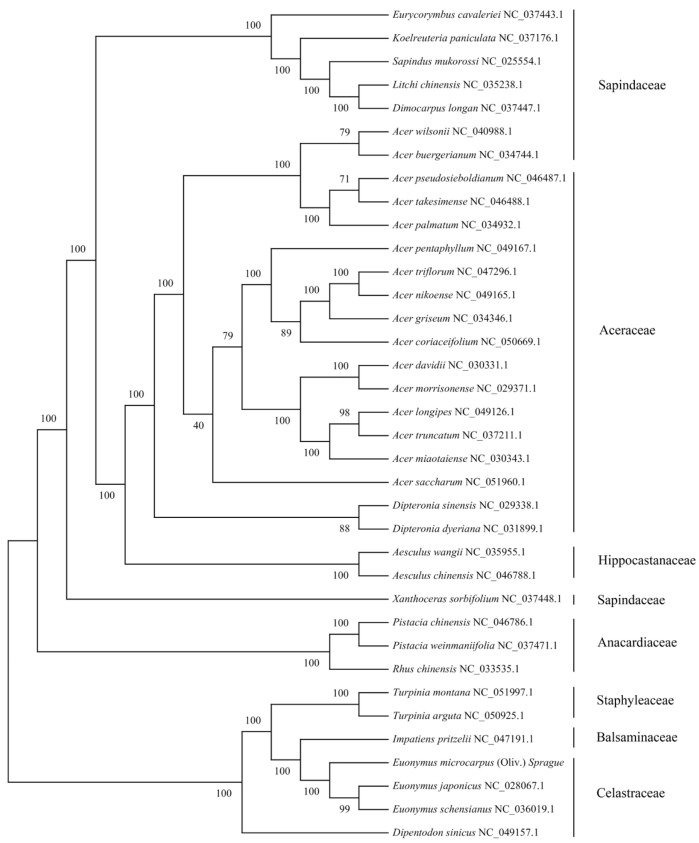
Phylogenetic tree of 36 complete cpDNAs constructed using the maximum likelihood (ML). The numbers above the branches represent the ML bootstrap values.

We selected cpDNAs of *E. microcarpus* and another 35 species within the family *Sapindaceae*, *Aceraceae*, *Hippocastanaceae*, *Sapindaceae*, *Anacardiaceae*, *Staphyleaceae*, *Balsaminaceae* and *Celastraceae,* to explore the genetic relationship between *E. microcarpus* and its relatives. Multiple alignments of all of the 36 cpDNAs was computed using MAFFT, then a maximum likelihood (ML) tree was determined using RAxML implementing the GTR-γ model. As shown in the Figure 4, all relationships were strongly supported by high bootstrap values ranging from 40 to 100.

The phylogenetic tree with most branches having high levels of support, shows two divergent clades. The first clade is formed by *Sapindaceae*, *Aceraceae*, *Hippocastanaceae*, *Sapindaceae* and *Anacardiaceae*, whereas the other is formed by *Staphyleaceae*, *Balsaminaceae* and *Celastraceae*. The phylogenetic analyses have established that three *Euonymus* species, *Turpinia montana*, *Turpinia arguta*, *Impatiens pritzelii* and *Dipentodon sinicus,* fell into the second clade. *Dipentodon sinicus* is located at the base of the clade. Subsequently, two *Turpinia* species and *Impatiens pritzelii* are separated. In addition, *E. japonicus* is sister to *E. schensianus*, and they are successively sister to *E. microcarpus* (Figure 4).

### 3.4. Comparative Analysis of the Euonymus cpDNA

**Figure 5:**
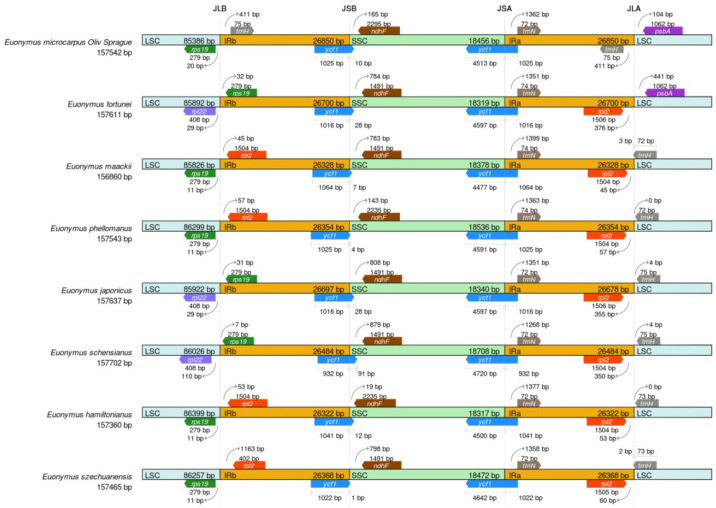
Border comparisons of the eight selected cpDNAs.

To advance our understanding of the cpDNAs of the genus *Euonymus*, further investigation was conducted to make critical comparisons of the IR/SSC and IR/LSC border positions in the eight selected *Euonymus* species, to access the degree of IR expansion or contraction among them. As shown in Figure 5, a large variability of the junction sites was observed in the eight cpDNAs, of which the LSC, IRa/b, and SSC regions have an average length of 86,001 bp, 26,513 bp and 18,441 bp, respectively. 

The *rps19* of *E. microcarpus*, *E. maackii*, *E. phellomanus*, *E. hamiltonianus* and *E. szechuanensis,* were located completely at the junction of the LSC. The *trnH-GUG* and *psbA* of *E. microcarpus* was located at the JLA junction. The trnH-GUG was completely found in the IRa region, while the *psbA* was completely located in the LSC region. The JLB junction of *E. fortunei*, *E. japonicus* and *E. schensianus* was positioned between the two genes, *rpl22* and *rps19.* In the meantime, the gaps present in the gene rps19 in IRb from the JLB junction point were 32, 31 and 7 bp, respectively. 

The *trnH-GUG* resided at the IRa and was 411 bp from the IRa/LSC border in *E. microcarpus*. In addition, the *trnH-GUG* and *rpl2* of *E. maackii*, *E. phellomanus*, *E. japonicus*, *E. schensianus*, *E. hamiltonianus* and *E. szechuanensis* were positioned at the junction of JLA. In the midst of them, there was a consolidation of *trnH-GUG* into the IRa region 3 and 2 bp in the respective cpDNAs of *E. maackii* and *E. szechuanensis*. In the meantime, the *trnH-GUG* of *E. phellomanus*, *E. japonicus*, *E. schensianus* and *E. hamiltonianus* was totally positioned in the LSC region. 

Furthermore, in the cpDNAs of all of the species, a complete copy of the functional *ycf1* gene crossed JSA, while a truncated copy of *ycf1* detected as a pseudogene fragment was located at JSB and very close to *ndhF*. The *ycf1* fragments exhibited a 10-, 28-, 7-, 4-, 28-, 91-, 12- and 1-bp-long expansion from the IRb to the SSC regions in the cpDNAs of *E. microcarpus*, *E. fortunei*, *E. maackii*, *E. phellomanus*, *E. japonicus*, *E. schensianus*, *E. hamiltonianus* and *E. szechuanensis*, respectively. Notably, the cpDNAs of *E. microcarpus*, *E. maackii*, *E. phellomanus*, *E. hamiltonianus* and *E. szechuanensis* contained rps19 s in the LSC regions, whereas that of the other three species contained *rpl22* in the LSC regions. All of the cpDNAs of all of the species possessed *trnN* in the IRa regions and the *trnN* were 1268–1399 bp away form the junction of JSA. 

**Figure 6:**
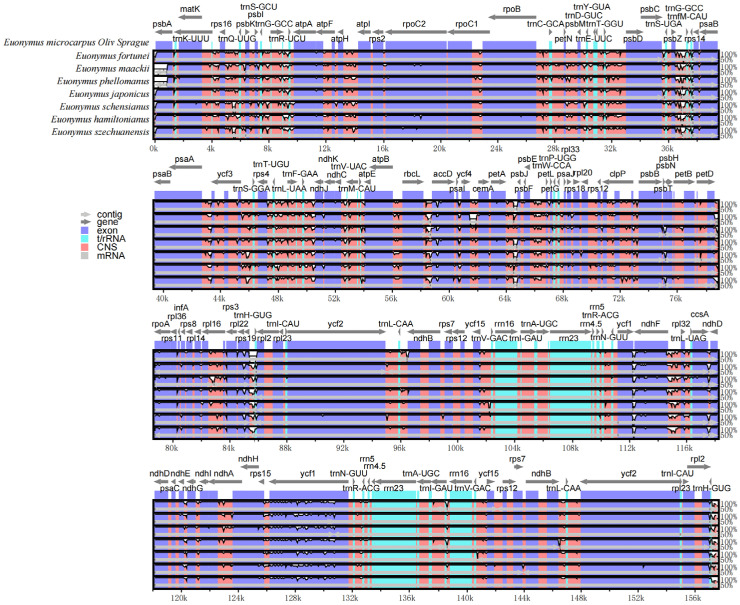
Visualization of the alignment of *E. microcarpus* and seven related species. The vertical scale indicates the percent identity ranging from 50 to 100%. Gray arrows and thick black lines above the alignment indicate the gene orientation.

The overall organization of the cpDNAs among the selected eight species assessed using the cpDNA of *E. microcarpus* as reference, showed a similarity of the structure and gene order. In the meantime, the level of divergence of the non-coding regions between the species selected was higher than that of the coding regions and the same thing happened in the comparison between the single copy regions and IRs (Figure 6). 

**Figure 7:**
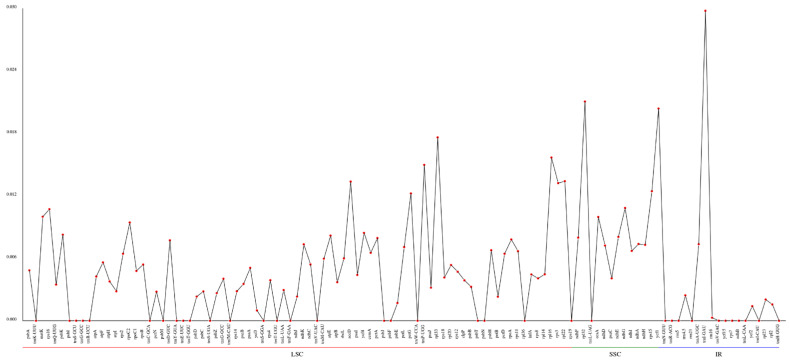
Comparative analysis of the gene nucleotide variability (pi) values of eight *Euonymus* species. The *X*-axis and *Y*-axis show the genes and the pi values, respectively.

The nucleotide diversity (pi) values of the selected eight cpDNAs, calculated within the slide window, ranged from 0 to 0.02976, with an average of 0.00471 (Figure 7). According to the data, three regions were highly variable, IR.*trnI-GAU*, SSC.*rpl32* and SSC.*ycf1*, with pi values higher than 0.02. The results showed low pi values, which suggests the cpDNA sequences are highly conserved at the sequence level throughout the genus.

Between *E. microcarpus* and each of *E. microcarpus*, *E. fortunei*, *E. maackii*, *E. phellomanus*, *E. japonicus*, *E. schensianus*, *E. hamiltonianus* and *E. szechuanensis*, the substitution rates of synonymous (Ks) and nonsynonymous (Ka) were determined to test for the adaptive selection (Table S5). These results suggest that four genes of cpDNA of *E. microcarpus*, *rpl16*, *ycf2-2*, *ycf1-2* and *atpF* with Ka/ks ratios as >1, underwent a positive selection in the comparisons of the *E. microcarpus,* versus the chosen species (Table S5). On the contrary, the remaining genes, with Ka/Ks < 1, underwent a negative selection, suggesting a slower evolution rate. 

## 4. Discussion

The plant cpDNA carries an average of 120 genes, including genes involved in the gene expression and genes related to photosynthesis [28]. The precise cpDNAs of *E. microcarpus*, a circular molecule of 157,542 bp displaying a characteristic quadripartite structure as with other angiosperm cpDNAs (Figure 1), 87 potential protein-coding, 38 transfer RNA (tRNA), and eight ribosomal RNA (rRNA) genes (Table 1). 

The GC content of the overall cpDNA is 37.22%, whereas that of the IR regions is higher than the LSC and SSC regions (Figure 2). The same thing happened in the cpDNAs of *E. fortunei*, *E. maackii*, *E. phellomanus*, *E. japonicus*, *E. schensianus*, *E. hamiltonianus* and *E. szechuanensis*, respectively [14,29]. The GC content is treated as an indicator of the local recombination rate and stability of the secondary structures [30].

The repeat sequences may raise the genetic diversity of the species and advance the cpDNA rearrangements. Generally, the cpSSRs, more easily obtained than the nuclear SSRs, are valuable genetic tools for addressing basic and applied plant biology questions, because of their codominant nature, high polymorphism and low substitution rate. Previous research detected 79 to 135 SSRs and 43 to 56 long repeats in the cpDNAs of six species of genus *Euonymus,* including *E. Japonicus*, *E. maackii*, *E. phellomanus*, *E. schensianus*, *E. hamiltonianus* and *E. Fortunei* [31], while the present study detected 282 SSRs and 63 long repeats in the cpDNA of *E. microcarpus*. Among the SSRs and the long repeats of the seven species of genus *Euonymus*, the mononucleotide SSRs were the majority, while the complement repeats were the minority. The SSR motifs could serve as potential molecular markers for the species identification, analysis of the population genetic and the genetic differentiation between individuals [31,32].

The phylogenetic analysis was conducted, based on the cpDNAs of 36 species within the order Sapindales. In the first clade, the cluster formed by three species within the family *Anacardiaceae*, including two species of the genus *Pistacia* and one species of the genus *Rhus*, diverged first, forming a sister relationship with a 100% bootstrap support. Then, *Xanthoceras sorbifolium*, belonging to the family *Sapindaceae*, diverged and formed a sister relationship to the family *Sapindaceae*, Aceraceae and *Hippocastanaceae* with a 100% bootstrap support. Having been cluster formed by the genus *Acer* and *Dipteronia*, the *Aceraceae* form a monophyletic cluster and is most closely related to the cluster of two *Aesculus* species. The results are consistent with current taxonomic classifications, showing the family Aceraceae and *Hippocastanaceae*, not distinct from the *Sapindaceae*, are in fact nested in the family *Sapindaceae* [33]. In the second clade, the phylogenetic tree fully supports that *E. microcarpus* is most closely associated to the cluster of *E. japonicus* and *E. schensianus* with a 100% bootstrap value. The genus *Euonymus* is monophyletic and is closely associated to *Impatiens pritzelii* and the cluster of two *Turpinia* species, successively, with high support scores. *Dipentodon sinicus* within *Celastraceae*, diverged first, forming a sister relationship to the family *Staphyleaceae*, *Balsaminaceae* and *Celastraceae*. This study showed that *Dipentodon sinicus* is not most closely related to three species of the genus *Euonymus* within *Celastraceae*. The systematic position of *Dipentodon sinicus* has been highly controversial since it was placed in the family *Celastraceae* [34]. Therefore, to better understand the phylogeny and evolution of the order Sapindales, more species are necessary. This study provides a valuable basis for future phylogenetic analyses of species within *Celastraceae*. 

The modification of the cpDNA size could be driven by the important evolutionary events, expansion and shrinkage of the IR region, which occur frequently, lead to fluxes in the LSC/IR junctions and the initiation of pseudogenes, gene duplication or a reversion of the duplicated genes to a single copy [35]. Previous studies have found that the contraction and expansion of IRs affect the evolution rate of the protein-coding genes in the subfamily *Pothoideae* in the family *Araceae* [36]. In this study, we compared the cpDNAs of eight *Euonymus* species. In the cpDNAs of angiosperm, the borders between the LSC/SSC and IR regions are highly and relatively conserved [37]. It is clear that the *rps19* of *E. microcarpus*, *E. maackii*, *E. phellomanus*, *E. hamiltonianus* and *E. szechuanensis,* were completely positioned in the LSC region at the junction of JLB, while, that of *E. japonicus*, *E. schensianus* and *E. fortune,* were located in the IRb region. It is obvious that the cpDNAs of *E. japonicus*, *E. schensianus* and *E. fortunei* showed expansions and contractions of the IRs and LSC, respectively, which is consistent with the findings of earlier reports [14]. The truncated copies of ycf1 were pseudogenized and observed at the junction of JSB in all of the eight selected *Euonymus* species. The results increase the current knowledge of the evolutionary patterns in the angiosperms.

## 5. Conclusions

In conclusion, the cpDNA of *E. microcarpus*, sequenced using an Illumina HiSeq X Ten system, displayed a typical quadripartite structure of most angiosperms. A large number of SSRs were detected, which could provide valuable information for designing exceedingly variable molecular markers for the population genetics and ecological and evolutional studies of *E. microcarpus*. Based on the cpDNAs of 36 species within the order Sapindales, the phylogenetic analysis showed a close relationship among *E. microcarpus*, *E. japonicus* and *E. schensianus*, suggesting that they are sister species. A comparison of the structure of the cpDNAs of eight *Euonymus* species was performed and it is obvious that there is a nucleotide variability of the junction sites and a higher divergence of non-coding regions, compared to coding regions. The obtained genetic resources and the results of the above analyses will facilitate future studies in the implementation of the effective conservation and management strategies, to help develop affordable and efficient genetic assays for the species identification of the genus *Euonymus*.

## Figures and Tables

**Table 1:** Annotated genes in the *E. microcarpus* cpDNA.

Category	Group of Genes	Name of Gene
Self-replication	Ribosomal RNA	*rrn4.5*^3^, *rrn5* ^3^, *rrn16* ^3^, *rrn23* ^3^
	Transfer RNA	*trnY-GUA*, *trnW-CCA*, *trnV-UAC 1*, *trnV-GAC* ^3^, *trnT-UGU*, *trnT-GGU*, *trnS-UGA*, *trnS-GGA*, *trnS-GCU*, *trnR-UCU*, *trnR-ACG* ^3^, *trnQ-UUG*, *trnP-UGG*, *trnN-GUU* ^3^, *trnM-CAU*, *trnL-UAG*, *trnL-UAA* ^1^, *trnL-CAA* ^3^, *trnK-UUU* ^1^, *trnI-GAU* ^1,3^, *trnI-CAU* ^3^, *trnH-GUG* ^3^, *trnG-GCC*, *trnG-GCC* ^1^, *trnfM-CAU*, *trnF-GAA*, *trnE-UUC*, *trnD-GUC*, *trnC-GCA*, *trnA-UGC* ^1,3^
	Small subunit of ribosome	*rps2*, *rps3*, *rps4*, *rps7* ^3^, *rps8*, *rps11*, *rps12* ^2,3^, *rps14*, *rps15*, *rps16*, *rps18*, *rps19*
	Large subunit of ribosome	*rpl2* ^1,3^, *rpl14*, *rpl16* ^1^, *rpl20*, *rpl22*, *rpl23* ^3^, *rpl32*, *rpl33*, *rpl36*
	RNA polymerase subunits	*rpoA*, *rpoB*, *rpoC1* ^1^, *rpoC2*
	Subunits of photosystem I	*psaA*, *psaB*, *psaC*, *psaI*, *psaJ*
	Subunits of photosystem II	*psbA*, *psbB*, *psbC*, *psbD*, *psbE*, *psbF*, *psbH*, *psbI*, *psbJ*, *psbK*, *psbM*, *psbN*, *psbT*, *psbZ*
Photosynthesis	Subunits of cytochrome	*petA*, *petB*^1^, *petD*^1^, *petG*, *petL*, *petN*
	Subunits of ATP synthase	*atpA*, *atpB*, *atpE*, *atpF* ^1^, *atpH*, *atpI*
	Large subunit of RuBisCO	*rbcL*
	Subunits of NADH	*ndhA* ^1^, *ndhB* ^1,3^, *ndhC*, *ndhD*, *ndhE*, *ndhF*, *ndhG*, *ndhH*, *ndhI*, *ndhJ*, *ndhK*
Other gene	Maturase	*matK*
	Envelope membrane protein	*cemA*
	Subunit of acetyl-CoA	*accD*
	C-type cytochrome synthesis gene	*ccsA*
	Protease	*clpP 2*
Unknown function	Conserved open reading frames	*ycf1* ^3^, *ycf2* ^3^, *ycf3* ^2^, *ycf4*, *ycf15* ^3^

^1^ Gene containing a single intron; ^2^ gene containing two introns; ^3^ two gene copies in the IRs.

**Table 2:** Genes with introns in the *E. microcarpus* cpDNA.

Gene	Region (bp)	Exon I (bp)	Intron I (bp)	Exon II (bp)	Intron II (bp)	Exon III (bp)
*atpF*	LSC	144 −	698	411 −		
*clpP*	LSC	69 −	624	291 −	848	231 −
*ndhA*	SSC	553 −	1168	539 −		
*ndhB*	IRB	777 −	685	756 −		
*ndhB*	IRA	777 +	685	756 +		
*petB*	LSC	6 +	766	642 +		
*petD*	LSC	8 +	723	475 +		
*rpl16*	LSC	9 −	1109	399 −		
*rpl2*	IRB	391 −	679	434 −		
*rpl2*	IRA	393 +	640	471 +		
*rpoC1*	LSC	435 −	818	1626 −		
*rps12*	IRB	232 −	-	26 +	546	114 +
*rps12*	IRA	114 −	-	232 −	546	26 −
*trnA-UGC*	IRB	38 +	799	35 +		
*trnA-UGC*	IRA	38 −	799	35 −		
*trnG-GCC*	LSC	23 +	771	49 +		
*trnI-GAU*	IRB	42 +	946	35 +		
*trnI-GAU*	IRA	42 −	946	35 −		
*trnK-UUU*	LSC	37 −	2502	35 −		
*trnL-UAA*	LSC	37 +	550	50 +		
*trnV-UAC*	LSC	39 −	676	37 −		
*ycf3*	LSC	126 −	739	228 −	734	153 −

+ Exon is transcribed counterclockwise in Figure 1; − exon is transcribed clockwise in Figure 1; - spliceosomal intron.

## Data Availability

The data used in our study have been submitted to the NCBI GenBank (accession number: ON611716). The related species and their GenBank accession numbers in this study are listed as follows: *Eurycorymbus cavaleriei* (NC_037443.1), *Koelreuteria paniculata* (NC_037176.1), *Sapindus mukorossi* (NC_025554.1), *Litchi chinensis* (NC_035238.1), *Dimocarpus longan* (NC_037447.1), *Acer wilsonii* (NC_040988.1), *Acer buergerianum* (NC_034744.1), *Acer pseudosieboldianum* (NC_046487.1), *Acer takesimense* (NC_046488.1), *Acer palmatum* (NC_034932.1), *Acer pentaphyllum* (NC_049167.1), *Acer triflorum* (NC_047296.1), *Acer nikoense* (NC_049165.1), *Acer griseum* (NC_034346.1), *Acer coriaceifolium* (NC_050669.1), *Acer davidii* (NC_030331.1), *Acer morrisonense* (NC_029371.1), *Acer longipes* (NC_049126.1), *Acer truncatum* (NC_037211.1), *Acer miaotaiense* (NC_030343.1), *Acer saccharum* (NC_051960.1), *Dipteronia sinensis* (NC_029338.1), *Dipteronia dyeriana* (NC_031899.1), *Aesculus wangii* (NC_035955.1), *Aesculus chinensis* (NC_046788.1), *Xanthoceras sorbifolium* (NC_037448.1), *Pistacia chinensis* (NC_046786.1), *Pistacia weinmaniifolia* (NC_037471.1), *Rhus chinensis* (NC_033535.1), *Turpinia montana* (NC_051997.1), *Turpinia arguta* (NC_050925.1), *Impatiens pritzelii* (NC_047191.1), *Euonymus japonicus* (NC_028067.1), *Euonymus schensianus* (NC_036019.1), *Dipentodon sinicus* (NC_049157.1).

## Supplementary Materials

The following supporting information can be downloaded at: https://www.mdpi.com/article/10.3390/genes13122352/s1, Table S1: Statistics of the synteny between the *E. microcarpus* and *E. szechuanensis* cpDNAs; Table S2: Codon usage of the *E. microcarpus* cpDNA from the RSCU tools; Table S3: Long repeat sequences in the *E. microcarpus* cpDNA; Table S4: Simple sequence repeats (SSRs) in the *E. microcarpus* cpDNA; Table S5: Ka/Ks ratios of the cp genes from *E. microcarpus* and seven related species.
